# Product development programs for neglected tropical diseases: A crucial role for expert meetings

**DOI:** 10.1371/journal.pntd.0005183

**Published:** 2017-02-23

**Authors:** Leonie Hussaarts, Kim van der Weijde, Pierre Dome, Elly Kourany-Lefoll, Jutta Reinhard-Rupp, Remco de Vrueh, on behalf of the Pediatric Praziquantel Consortium

**Affiliations:** 1 Lygature, Utrecht, The Netherlands; 2 Global Health R&D Department, Coinsins, Switzerland, being part of the biopharma business of Merck KGaA, Darmstadt, Germany; Universidad Nacional Autónoma de México, MEXICO

## The challenge

Development of new drugs and vaccines for neglected tropical diseases (NTDs) is unattractive from a market perspective due to the lack of sufficient financial incentives and low return on investment. To address the health burden of NTDs in low- and middle-income countries, research and development (R&D) programs for this group of diseases are increasingly handled by public—private partnerships (PPPs) [1,2].

In the WHO report on Priority Medicines (2013), PPPs for health are defined as “any informal or formal arrangement between one or more public sector entities and one or more private sector entities created in order to achieve a public health objective or to develop a health-related product or service for the public good” [3]. The partners in a PPP share development risks and integrate (financial) resources, technical facilities, and knowledge into a collective action. Depending on their mission and the objectives, PPPs cluster into one of three categories: early stage R&D (precompetitive and proof-of-concept), product development (PD), or product access [4].

PPPs face a number of challenges regardless of their health objective. In short, these challenges include time lines and sustainability; the role of small and medium-sized enterprises (SMEs); consortium leadership; the role of the central entity; intellectual property; and performance management (discussed in [3]). On top of these, PD-PPPs for control and elimination of NTDs face additional challenges [5], such as cultural and geographical differences, education of health care workers, regulatory affairs, and access to medicines. These factors all have a strong impact on the success and sustainability of a PPP.

Based on a systemic review of ten PD-PPPs for NTDs, De Pinho Campos and colleagues propose a model of collaboration that requires capitalization of expertise and early engagement of key stakeholders, including policy leaders. Such a model proved to be beneficial for building up understanding and support during the regulatory process and to ensuring long-term sustainability [6]. Expert meetings provide an excellent vehicle to support and strengthen such a model of collaboration. Designed outside regular project meetings to obtain an expert opinion on how to advance R&D programs, expert meetings are standard practice in the pharmaceutical industry. The Pediatric Praziquantel (PEDPZQ) Consortium, a PD-PPP that aims to develop and provide a suitable PEDPZQ formulation, has systematically organized expert meetings tailored to the specific needs of a particular phase of the R&D process. By sharing experiences from the PEDPZQ Consortium, this symposium highlights the crucial importance of expert meetings in PD programs for NTDs.

## Tutorial

### What is an expert meeting?

Expert meetings provide an opportunity to consult key opinion leaders or technical experts during the course of R&D programs. In the pharmaceutical industry, such meetings are frequently organized to bridge the gap between science and the clinic, facilitating the translation of knowledge into therapies. For example, experts may be consulted to review a portfolio, to obtain insight on how to advance R&D programs, or to determine the likelihood of success for a potential treatment [7]. In the context of PPPs for NTDs, expert meetings serve an additional purpose: they aim to avoid unforeseen pitfalls by closing the gap between the partnership’s and the target communities’ definition of an appropriate and affordable treatment.

In the context of the PEDPZQ Consortium, this meant recruiting external experts from the scientific and target communities to form an international scientific expert panel. The expertise of the panel members included (but was not limited to) pediatric formulations, epidemiology, worm control, or regulatory affairs. During the expert meetings (three had been organized at the time of writing), Consortium plans were presented and reflected upon by the expert panel and representatives from all Consortium partners and the Consortium Board, with the aim to achieve integration of R&D and applicability. The expert panel provided essential scientific, clinical, regulatory, and cultural advice, from which the Core Project Team (Box 1) subsequently distilled the most important messages and formulated concrete actions. These were then presented to the Consortium Board for final accord. Indeed, the insights were regarded as critical to the Consortium’s decision-making process. The meetings also allowed the Consortium to build a network that will foster local credibility and trust, which will be of crucial importance for the implementation phases of the R&D program.

**Box 1: The PEDPZQ Consortium**

The PEDPZQ Consortium was founded in 2012 to contribute to reduce the global disease burden of schistosomiasis by addressing the medical need of infected preschool-aged children who are currently devoid of treatment. The Consortium is developing a new PEDPZQ formulation with proven safety and therapeutic efficacy against *Schistosoma* worms. The child-friendly formulation consists of small, orodispersible tablets with an acceptable taste. After having successfully completed the preclinical phase, the program has progressed into the clinical development stage.

**Fig 1:**
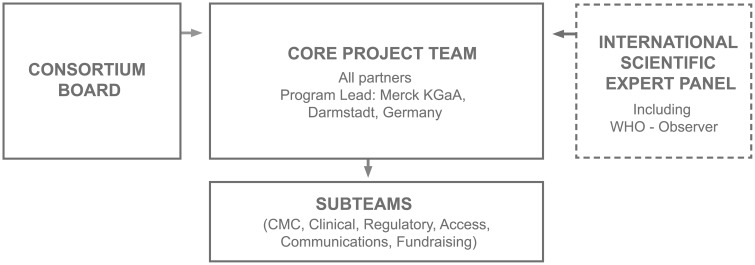
Governance structure of the PEDPZQ Consortium.

At the time of writing, the Consortium’s core team was comprised of Astellas Pharma (Tokyo, Japan), Merck KGaA (Darmstadt, Germany), Simcyp (United Kingdom), Farmanguinhos/FIOCRUZ (Rio de Janeiro, Brazil), Lygature (formerly Top Institute Pharma; Utrecht, the Netherlands), the Schistosomiasis Control Initiative (United Kingdom), and the Swiss Tropical & Public Health Institute (Basel, Switzerland). These partners were brought together based on their expertise in formulation development, process development and scale-up, nonclinical and clinical development, pharmacokinetic modelling, tropical disease epidemiology, regulatory affairs, NTD control, PPP coordination and drug R&D management. The core project team is responsible for designing, driving, and implementing the development of the PEDPZQ formulation and is overseen by a consortium board, comprised of executive members of the partner organizations. As discussed in this Symposium, the Consortium frequently consults an international scientific expert panel (Fig 1).

### Which topics are typically discussed during an expert meeting?

Unique to the PEDPZQ Consortium, expert meetings were implemented early in the development process and prior to every critical decision-making step in the program. As an outcome of this systematic approach, the topics that were discussed depended heavily on the specific phase of the project (Table 1). Although every NTD R&D program is unique, it is possible to identify topics that would be of crucial importance to any NTD program. These topics include developing a target product profile, the clinical trial study design, and the regulatory strategy. It also proved of crucial importance to regularly liaison with the WHO, which will eventually play a critical role in the regulatory/drug approval process for the prequalification of the new formulation and in the development of an access and delivery plan.

**Table 1:** Expert meetings organized by the PEDPZQ Consortium.

	Expert Meeting 1	Expert Meeting 2	Expert Meeting 3
Title	Target product profile	Clinical design	Regulatory strategy
Objective	To seek experts’ feedback on the target characteristics of the drug to be developed.	To seek advice regarding the design of the clinical development program.	To build knowledge and trust between the Consortium and endemic country regulatory experts.
Experts attended	9	10	12
Continents represented by experts	Europe (5), Africa (2), South America (1), Asia (1)	Europe (3), Africa (4), South America (2), North America (1)	Europe (2), Africa (10)
Topics discussed	Apart from a general introduction of the Consortium and the project, the following topics were discussed during a series of plenary sessions: Problem statement in young children Target population and therapeutic indication Potential pediatric formulations suitable for use in young children living in hot and humid tropical settings Unmet needs and efficacy and safety profile. Pharmaco-economics Manufacturing profile Regulatory strategy	Apart from a general introduction of the Consortium and the project during a plenary session, the following clinical studies were discussed during a series of break-out sessions: Taste study Phase I Bioavailability study Phase II pediatric exploratory study Phase III confirmatory study	Apart from a general introduction of the Consortium and the development program by Consortium representatives, experts outlined the following: Regulatory procedures available at the WHO Status of regulatory harmonization in Africa The current status of the regulatory environment in his/her country (seven in total) During a series of break-out sessions, in-depth discussions focused on the following issues: Clinical Trial Application and Ethics Committee process and approval Regulatory approach
Most important recommendations and outcomes	Target population of infants, toddlers, and children from three months until six years was confirmed A single dose administration once a year (in highly endemic regions) was confirmed Better understanding of PK/PD of praziquantel is needed to plan clinical development of PEDPZQ formulation in relation to current commercial formulation A solid formulation was preferred over a liquid one Shelf life of the new pediatric formulation should preferably be 24–36 months Sweetener or sugar (but not flavor) could be added to the tablet to mask bitterness Two formulation options were discussed in detail: Solid mini-tablets: key concerns raised were a.o. inability of infants to swallow a mini-tablet, and use of dosing device is too complicated in the field Oro-dispersible tablets: key concerns raised were drug loading, production cost, and need for in-use stability studies Investigate with potential procurers how much on top of the cost of current PZQ they would be willing to pay for a pediatric product Evaluate the potential “first” registration in Brazil, an endemic country with expertise and seen as a valid reference country for other countries As the drug will be administered in mass, including infected and healthy people, data in healthy volunteers are important As a risk mitigation action: develop both a racemate PZQ and a L-enantiomer PZQ formulation	Using a draft synopsis for each study and a list of pre-defined questions to guide the discussion, recommendations were given related to age group, treatment arms, diagnostic method, inclusion criteria, sample size, study location, study methodology, safety tests, and dosing regimen A set of success criteria was defined: Child-appropriate formulation that is easily administered, safe, efficacious, and accepted by preschool-aged children New PK/PD data in infected children of all ages Finding the correct dose(s) for young children Dosing guidelines derived from studies in children Cure rate equivalent or higher than the current PZQ Curable, no unpleasant smell, safe (side effects similar or less than the current formulation) Time to market, affordable costs of treatment, and engagement with regulatory authorities	Using a briefing document and a list of pre-defined questions to guide the discussion, recommendations were given related to the following issues: Regulatory registration options that were presented as well as alternatives Ensuring joint agreement on the clinical development plan Alignment with other countries Future engagement Specifics around the registration process GMP inspection is being consolidated more and more, but not everywhere The WHO prequalification approach is preferred by the participating countries The participants emphasized the value of this meeting with respect to establishing a working relationship between the Consortium and the regulators in order to address an unmet medical need for the young infected children in their country

Abbreviations: a.o., among others; GMP, good manufacturing practice; PK/PD, pharmacokinetics/pharmacodynamics

### Why are expert meetings crucial to the success of NTD programs?

While any R&D program will benefit from expert meetings, they are particularly essential for programs directed at low- and middle-income countries. The leaderships of such programs are frequently based in more economically developed countries, with limited knowledge of the disease, the needs and limitations of the target community, and the local regulatory environment. Examples from the PEDPZQ Consortium illustrate why expert meetings are crucial to the success of NTD programs.

The first expert meeting organized by the PEDPZQ Consortium illustrates how “local” knowledge can crucially contribute to the success of the program. During this meeting, the acceptability of the current target product profile was discussed (Table 1). While the Consortium had initially planned to add flavor to the tablet formulation as a taste masking agent, African physicians advised against it because the target community would be largely unfamiliar with the added flavor. The experts suggested using only sweetener or sugars to mask the bitter taste of the tablet, which was then implemented into the R&D program.

During the second expert meeting, the complete clinical development program was discussed. As described in Table 1, the planned program consisted of a taste study and four clinical trials (two Phase I bioavailability studies, a Phase II exploratory trial, and a Phase III confirmatory study). The clinical experts provided detailed recommendations for improving the study designs and identified several criteria imperative for success. They also emphasized the need for pharmacokinetic/pharmacodynamic (PK/PD) data and dosing guidelines derived from studies in children, to ensure correct dosing of infected children during the Phase II and III trials.

During the third expert meeting focusing on regulatory activities, regulatory experts from seven sub-Saharan African (SSA) countries provided critical insights into the national and regional SSA pharmaceutical regulatory environment. The group of experts indicated that the WHO prequalification collaborative approach for registration of the novel drug was preferred. This procedure, implemented in 2013, allows fast registration of drugs in the SSA participating countries following their prequalification by WHO [8]. In addition, all experts valued the meeting with respect to establishing a working relationship with the Consortium in order to address the unmet medical need for the young infected children in their countries.

## How to organize an expert meeting?

It is important to understand that each PD-PPP is unique, and organizational aspects should therefore be tailored to the specific needs of the project at stake. Four key elements are addressed below that the PEDPZQ Consortium considered instrumental, which may help to guide other PD-PPPs in the organization of their expert meetings.

### Timing

As described above, all three expert meetings resulted in modifications of established R&D plans and strategies. It is therefore critical to time the meetings in such a way that adjustments can be easily made without lost investments or delays. For example, discussions on regulatory strategy and related activities may influence the design of the clinical program, which is why it is important to think two steps ahead.

### Selection of experts

The participants at the PEDPZQ Consortium’s expert meetings were selected because they were considered key opinion leaders in their respective fields. Of note, many had had prior interaction with one of the consortium partners on related topics. It proved of particular importance to engage experts from the target community who can contribute “local” knowledge. Relatedly, it was imperative to engage a varied selection of African countries for programs directed at African communities, as each country within SSA typically displays a heterogeneous pharmaceutical and health care environment when compared amongst each other [9].

### Meeting facilitation

In case of the PEDPZQ Consortium, the role of the experts as an advisory one was addressed and clearly explained in the invitation letter or E-mail. In addition, the experts were appropriately informed about the project and the topics to be discussed ahead of the meeting. They received an agenda and a briefing book, with a description of the Consortium, the project status, the scope and objective of the meeting, a list of specific questions for the experts, and related annexes (i.e., synopses of the clinical studies). This allowed sufficient time for the experts to prepare for the meeting upfront and identify any issues unforeseen by the Consortium. For one of the expert meetings, given the participation of francophone experts, the agenda and briefing book were translated into French, and dedicated interpreters were present at the meeting itself.

During the meeting, the expression of various viewpoints was encouraged, especially when it concerned differences between West and East Africa.

Finally, the experts were given the opportunity to provide their input on the draft meeting minutes, which were made available to them. They are also kept up to date as the project evolves, through biannual newsletters and personal contact with Consortium members.

### Expert remuneration

To further ensure the independence and commitment of the experts, expert remuneration was restricted to cover travel and lodging costs only. The benefits that expert meetings bring to an NTD development project in terms of increased efficiency, effectiveness, and the avoidance of unnecessary costs generally outweighs the costs of its organization. Therefore, we advise that a PD-PPP does not forgo an opportunity for an expert meeting on the basis of lack of funding alone.

## Concluding remarks

By sharing experiences from the PEDPZQ Consortium, this article highlights the crucial importance of expert meetings in NTD PD programs and provides guidance to PD-PPPs on how to organize such meetings.

As for the PEDPZQ Consortium, the team is well on its way developing a PEDPZQ formulation for preschool-age children. The Phase II clinical trial is currently ongoing, and the Phase III clinical trial is scheduled for 2017–2018. Importantly, the development and registration of a novel PZQ formulation is only part of the equation. Post-registration implementation studies, capacity to monitor safety and efficacy under use conditions, clear access and product costing strategies, and adequate preparedness of health care workers are only a few of the many activities that will determine the ultimate success of the product. Indeed, it is important to continue to organize expert meetings and to engage with relevant stakeholders, including the WHO and local health authorities, to avoid unforeseen pitfalls that may jeopardize the implementation of a suitable PZQ formulation for preschool-aged children.

**Key learning points**

- R&D programs for NTDs are increasingly handled by PPPs that integrate resources, technical facilities, and knowledge of public and private partners into a collective action to develop and deliver safe and affordable treatments.
- Expert meetings help to avoid pitfalls in R&D programs for NTDs by closing the gap between science, technology, and the target community.
- Expert meetings should be organized systematically and tailored to the objectives of each individual phase of the R&D program.
- It is important to time expert meetings in such a way that adjustments can be easily made without lost investments or delays.
